# The Influence of Telomere-Related Gene Variants, Serum Levels, and Relative Leukocyte Telomere Length in Pituitary Adenoma Occurrence and Recurrence

**DOI:** 10.3390/cancers16030643

**Published:** 2024-02-02

**Authors:** Greta Gedvilaite, Loresa Kriauciuniene, Arimantas Tamasauskas, Rasa Liutkeviciene

**Affiliations:** 1 Laboratory of Ophthalmology, Neuroscience Institute, Lithuanian University of Health Sciences, Medical Academy, Eiveniu 2, LT-50161 Kaunas, Lithuania; loresa.kriauciuniene@lsmuni.lt (L.K.); rasa.liutkeviciene@lsmuni.lt (R.L.); 2 Department of Neurosurgery, Lithuanian University of Health Sciences, Medical Academy, Eiveniu 2, LT-50161 Kaunas, Lithuania; arimantas.tamasauskas@lsmuni.lt

**Keywords:** Pituitary adenoma, *TNKS2*, *CTC1*, *ZNF676*, *TERF1*, *TERF2*

## Abstract

**Simple Summary:**

The study examined 130 pituitary adenoma (PA) patients and 320 healthy individuals, analyzing DNA samples. Real-time PCR and ELISA assessed genetic variations, telomere lengths, and serum proteins. Significant associations were found: *TERF1* rs1545827 CT + TT genotypes decreased PA occurrence odds, while *TNKS2* rs10509637 GG genotype increased the odds. Gender-specific patterns emerged: females with *TERF1* rs1545827 CC + TT had lower odds; also males with *TERF1* rs1545827 T allele showed decreased odds. *TNKS2* rs10509637 AA genotype increased odds in both genders and in PA recurrence. PA patients had elevated TERF2 and decreased TERF1 serum levels, longer telomeres, and *TERF1* rs1545827 T allele associated with reduced telomere shortening odds. Gender-specific genetic effects were observed, implicating *TERF1* and *TNKS2* in telomere regulation and PA susceptibility.

**Abstract:**

In this study, we examined 130 patients with pituitary adenomas (PAs) and 320 healthy subjects, using DNA samples from peripheral blood leukocytes purified through the DNA salting-out method. Real-time polymerase chain reaction (RT-PCR) was used to assess single nucleotide polymorphisms (SNPs) and relative leukocyte telomere lengths (RLTLs), while enzyme-linked immunosorbent assay (ELISA) was used to determine the levels of TERF1, TERF2, TNKS2, CTC1, and ZNF676 in blood serum. Our findings reveal several significant associations. Genetic associations with pituitary adenoma occurrence: the *TERF1* rs1545827 CT + TT genotypes were linked to 2.9-fold decreased odds of PA occurrence. Conversely, the *TNKS2* rs10509637 GG genotype showed 6.5-fold increased odds of PA occurrence. Gender-specific genetic associations with PA occurrence: in females, the *TERF1* rs1545827 CC + TT genotypes indicated 3.1-fold decreased odds of PA occurrence, while the *TNKS2* rs10509637 AA genotype was associated with 4.6-fold increased odds. In males, the presence of the *TERF1* rs1545827 T allele was associated with 2.2-fold decreased odds of PA occurrence, while the *TNKS2* rs10509637 AA genotype was linked to a substantial 10.6-fold increase in odds. Associations with pituitary adenoma recurrence: the *TNKS2* rs10509637 AA genotype was associated with 4.2-fold increased odds of PA recurrence. On the other hand, the *TERF1* rs1545827 CT + TT genotypes were linked to 3.5-fold decreased odds of PA without recurrence, while the *TNKS2* rs10509637 AA genotype was associated with 6.4-fold increased odds of PA without recurrence. Serum TERF2 and TERF1 levels: patients with PA exhibited elevated serum TERF2 levels compared to the reference group. Conversely, patients with PA had decreased TERF1 serum levels compared to the reference group. Relative leukocyte telomere length (RLTL): a significant difference in RLTL between the PA group and the reference group was observed, with PA patients having longer telomeres. Genetic associations with telomere shortening: the *TERF1* rs1545827 T allele was associated with 1.4-fold decreased odds of telomere shortening. In contrast, the *CTC1* rs3027234 TT genotype was linked to 4.8-fold increased odds of telomere shortening. These findings suggest a complex interplay between genetic factors, telomere length, and pituitary adenoma occurrence and recurrence, with potential gender-specific effects. Furthermore, variations in *TERF1* and *TNKS2* genes may play crucial roles in telomere length regulation and disease susceptibility.

## 1. Introduction

The pituitary adenoma (PA) is the third most common primary brain tumor, accounting for 14.1% of all such tumors [1]. Although PAs are generally characterized as non-cancerous [2], they can exhibit invasive tendencies, potentially leading to complications such as hypopituitarism and visual field impairment resulting from the compression of adjacent structures. Surgical intervention, primarily in the form of transsphenoidal surgery, is the preferred initial treatment for PAs [3]. However, it is important to note that despite surgical resection being the primary treatment, up to one-third of cases may experience recurrence, affecting both nonfunctional and functional adenomas. Therefore, identifying the risk of recurrence holds significant importance in guiding follow-up and adjuvant therapy decisions [1]. As we delve into the associations of PA and the importance of identifying their risk of recurrence, it is vital to consider the role of telomeres.

Telomeres are nucleoprotein complexes located at the end of eukaryotic chromosomes. Telomere length is known to shorten with age, and progressive telomere shortening can lead to somatic cell aging, apoptosis, or oncogenic transformation, all of which can affect a person’s health and life expectancy [4]. Shorter telomeres are associated with an increased incidence of disease and poorer survival rates [5]. Telomeres are closely associated with a protein complex called shelterin [6]. This shelterin complex serves to protect chromosomes from end-joining and breakage by forming unique t-loop structures [7]. These t-loop structures cover the ends of chromosomes, preventing them from being recognized as breaks in the double-stranded DNA chain, thereby preserving the stability and integrity of the chromosomes [8]. The CST complex inhibits telomerase, rendering it inactive, and it promotes the synthesis of the lagging strand by binding to single-stranded DNA [9]. Telomeric repeat binding factor 1 (TERF1) and telomeric repeat binding factor 2 (TERF2) act as negative regulators of telomere length, acting as telomerase inhibitors [10]. Overexpression of TERF1 and TERF2 can lead to telomere shortening, while reduced expression can lead to telomere elongation [11]. Tankyrase 2 (TNKS2) can be associated with longer telomeres in tumor cells when its expression is upregulated, suggesting that TNKS2 may promote tumor development and function as an oncogene [12]. The mechanisms linking telomere replication complex component 1 (CTC1) to leukocyte telomere length are apparently due to the gene’s membership in the CST complex [13]. The CST complex plays a vital role in preserving genome stability. The potential to explore small molecule inhibitors targeting shelterin and CST is being further examined for their potential in the therapeutic management of related diseases [14].

ZNF676 is a transcriptional regulator that holds intriguing implications for telomere homeostasis in humans. Telomere dysfunction is a well-recognized contributor to cancer development and is marked by the potential for genomic instability when coupled with a loss of cell cycle control [15]. However, the precise mechanisms through which ZNF676 influences telomere length remain unclear [16].

Theoretically, ZNF676 can modify telomere length in two ways. Firstly, direct binding to DNA can alter the expression of genes involved in telomere maintenance, and through interactions with proteins, it can also influence the post-translational signaling of these genes. Secondly, there is evidence that single-stranded telomeric DNA can fold into a structure known as a G-quadruplex, which, at the 3′ end of telomeres, can inhibit telomere elongation by telomerase [13]. Changes in telomere length are closely related to oncogenesis. Thus, we aimed to investigate whether telomere-associated genes and proteins are associated with the occurrence of pituitary adenomas. Additionally, we explored the potential impact on pituitary adenoma recurrence.

## 2. Materials and Methods

The study was conducted in the Laboratory of Ophthalmology, Lithuanian University of Health Sciences. Kaunas Regional Biomedical Research Ethics Committee approved the study (Approval number: BE-2-47, dated 25 December 2016). All participants were introduced to the structure and objectives of the present study before the execution. An Informed Consent Form was obtained from all subjects involved in the study.

### 2.1. Study Group

Study group I: patients with pituitary adenoma (*n* = 130). The PA group included PAs diagnosed and confirmed via magnetic resonance imaging (MRI), patients with good general health, aged 18 years and above, and with the absence of other tumors. The PA group was divided into subgroups by relapse. In our investigation, we observed that out of the total cohort, 38 patients from the Lithuanian population experienced tumor recurrence, while 92 patients did not exhibit relapse during the follow-up period. We acknowledge the significance of elucidating further details about the subgroup experiencing tumor recurrence to facilitate a more comprehensive understanding of the genetic associations and underlying mechanisms driving disease progression in patients with PAs within the Lithuanian population. Relapse was diagnosed if the enlargement of a residual tumor or a new growth was noticed and documented on a follow-up MRI, as previously described [17].

Study group II: healthy subjects (*n* = 320). The healthy control group consisted of age- and gender-matched subjects having good general health.

### 2.2. DNA Extraction

The DNA was extracted from peripheral venous blood samples (leucocytes) collected in 200 µL test tubes utilizing the silica-based membrane technology using a genomic DNA extraction kit (GeneJET Genomic DNA Purification Kit, Thermo Fisher Scientific, Vilnius, Lithuania), based on the manufacturer’s recommendations.

### 2.3. Genotyping

Single nucleotide polymorphisms of *TNKS2* rs10509639 and rs10509637, *CTC1* rs3027234, *ZNF676* rs412658, *TERF1* rs10107605 and rs1545827, *TERF2* rs251796 were carried out using the real-time polymerase chain reaction (RT-PCR) method. TaqMan^®^ Genotyping assays were used to determine SNPs (Applied Biosystems, Waltham, MA, USA; Thermo Fisher Scientific, Inc., Waltham, MA, USA) C__29498647_20, C__30418896_20, C__15770320_10, C__11463190_10, C___1869856_10, C___1869846_10, and C____706068_10 according to the manufacturer’s protocols using a StepOne Plus software 2.3 (Applied Biosystems). The repetitive analysis of 5% randomly chosen samples was performed for all five SNPs to confirm the same rate of genotypes from initial and repetitive genotyping.

### 2.4. Relative Leukocyte Telomere Length Measurement

Relative leukocyte telomere lengths (RLTLs) in PA and reference groups were studied. RLTL was measured using the quantitative real-time PCR method, as previous described [18]. The amounts of telomere DNA fragments and the reference gene albumin were determined in 2 replicates. We performed RT-PCR to determine the relative length of leukocyte telomeres using a real-time PCR multiplier, StepOne Plus (Applied Biosystems, USA). The reference DNA was a mixture of two randomly selected test DNA samples. Positive control: DNA isolated from a commercial human cell line 1301 with an extra-long telmere (Sigma Aldrich, St. Louis, MO, USA).

Primers:

Telg 5′-ACA CTA AGG TTT GGG TTT GGG TTT GGG TTT GGG TTA GTG T-3′

Telc 5′-TGT TAG GTA TCC CTA TCC CTA TCC CTA TCC CTA TCC CTA ACA-3′

Albd 5′-GCC CGG CCC GCC GCG CCC GTC CCG CCG GAA AAG CAT GGT CGC CTG TT-3′

Albu 5′-CGG CGG CGG GCG GCG CGG GCT GGG CGG AAA TGCTGC ACA GAA TCC TTG-3′

Two different methods are available for the analysis of real-time quantitative PCR data: absolute quantitative analysis and relative quantitative analysis. In our study, we opted for relative quantitative data analysis, as recommended by BioRad Laboratories (Hercules, CA, USA) in 2006.

To determine the relative telomere length (T/S) of peripheral blood leukocytes, we followed the relative analysis method devised by Livak in 2001 (T/S = 2^−ΔΔCt^) [19], after establishing the efficiency of PCR amplification. This approach is appropriate when the amplification efficiency of the telomere fragments and the albumin gene is high (within the range of 90–105%) and the difference in efficiency between them does not exceed 5%, as outlined by BioRad Laboratories in 2006.

The ΔCt value for each sample is computed by finding the disparity between the Ct value of the tested telomere fragments and the Ct value of the reference albumin gene:

ΔCt = Ct (telomere fragments) − Ct (reference albumin gene)

The ΔΔCt value characterizes the distinction between the ΔCt value of the test sample and the ΔCt value of the reference sample, which, similar to the test samples, has a concentration of 20 ng/μL:

ΔΔCt = Ct (test sample) − Ct (reference sample)

### 2.5. Serum Levels Measurement

**Figure 1:**
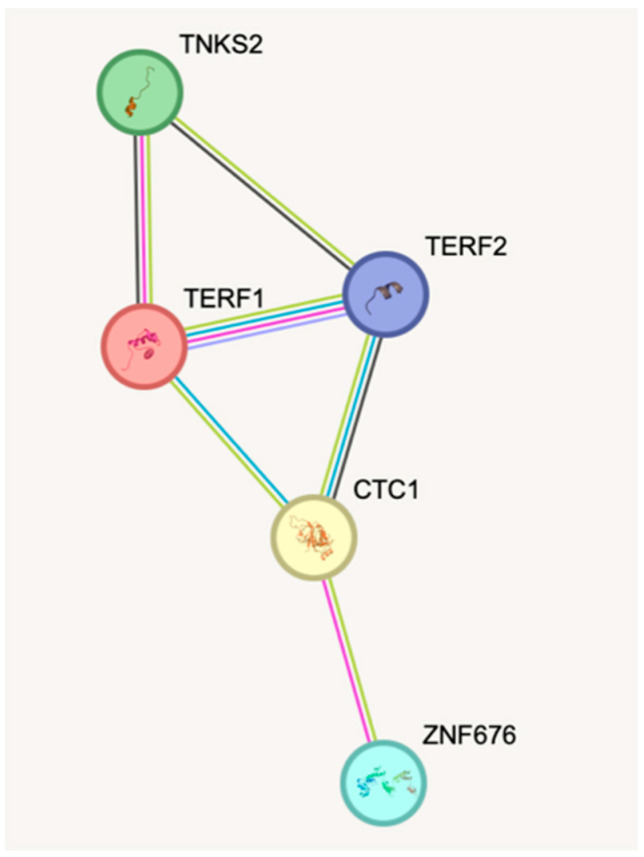
TNKS2, CTC1, ZNF676, TERF1, and TERF2 protein-protein connections.

In our study, ELISA Kits for Human Tankyrase 2 (TNKS2), CST Complex Subunit CTC1 (CTC1), Zinc Finger Protein 676 (ZNF676), telomeric repeat-binding factor 1 (TERF1), and telomeric repeat binding factor 2 (TERF2) from Abbexa, a manufacturer based in Cambridge, UK, were employed, utilizing sandwich enzyme-linked immunosorbent assay (ELISA) technology. Each kit features a 96-well plate with a pre-coated antibody. The HRP enzymatic reaction is quantified using TMB substrate, resulting in a blue product, which turns yellow after adding an acidic stop solution. The yellow color intensity corresponds to the bound protein’s concentration. Optical density (OD) is measured at 450 nm in a microplate reader to calculate the protein’s concentration, specifically in blood serum. Protein–protein connections are shown in Figure 1.

### 2.6. Study Characteristics

The study included 450 subjects divided into two groups: a reference group (*n* = 320) and patients with pituitary adenoma group (*n* = 130). The reference group was adjusted by gender and age to the PA group (*p* = 0.124; *p* = 0.620, respectively). Relative leukocyte telomere length (RLTL) was determined in 100 subjects with pituitary adenoma and 320 healthy subjects. Significant differences between the reference and pituitary adenoma groups were found between relative leukocyte telomere length (*p* < 0.001). The demographic data of the study subjects are presented in Table 1.

### 2.7. Statistical Analysis

The demographic characteristics data were compared between the reference and PA groups using the Pearson chi-square test, Student’s t-test, and Mann–Whitney U-test. The frequencies of *TERF1* rs1545827 and rs10107605, *TNKS2* rs10509637 and rs10509639, *TERF2* rs251796, *ZNF676* rs412658, and *CTC1* rs3027234 genotypes and alleles are presented in percentages. Binary logistic regression analysis was performed to evaluate selected SNP associations with PA occurrence. This was estimated considering inheritance models and genotype combinations (codominant, dominant, recessive, overdominant, and additive genetic models), giving an OR with a 95% confidence interval (CI). The Akaike information criterion (AIC) was evaluated in selecting the best inheritance model, with the lowest value indicating the most appropriate model. A nonparametric Mann–Whitney U-test was used to compare different groups when the data distribution was not normal. To test the statistical hypotheses, we chose a significance level of 0.05. A statistically significant difference was found when the *p*-value was 0.05. Statistical analysis was performed using the SPSS/W 29.0 software (Statistical Package for the Social Sciences for Windows, Inc., Chicago, IL, USA).

## 3. Results

The frequencies of genotypes and alleles for the following single-nucleotide polymorphisms (SNPs) were analyzed within the study groups: *TERF1* rs1545827, *TERF1* rs10107605, *TNKS2* rs10509637, *TNKS2* rs10509639, *TERF2* rs251796, *ZNF676* rs412658, and *CTC1* rs3027234.

For *TERF1* rs1545827 (CC, CT, and TT), we observed a statistically significant difference between the PA and reference groups, with frequencies of 62.3%, 31.5%, and 6.2% in PA, respectively, compared to 37.2%, 50.6%, and 12.2% in the reference group (*p* < 0.001). Furthermore, the T allele was less frequent in the PA group, accounting for 21.9% compared to 37.5% in the reference group (*p* < 0.001).

Similarly, for *TERF1* rs10107605 (AA, AC, and CC), we found a statistically significant difference between the PA and reference groups, with frequencies of 90.8%, 9.2%, and 0.0% in PA, respectively, compared to 80.9%, 13.1%, and 5.9% in the reference group (*p* = 0.007). The C allele was also less frequent in the PA group, accounting for 4.6% compared to 12.5% in the reference group (*p* < 0.001).

For *TNKS2* rs10509637 (AA, AG, and GG), a significant difference was observed between the PA and reference groups, with frequencies of 51.6%, 31.5%, and 16.8% in PA, respectively, compared to 68.5%, 28.1%, and 3.4% in the reference group (*p* < 0.001). The G allele was more frequent in the PA group, accounting for 32.7% compared to 17.5% in the reference group (*p* < 0.001). These results are summarized in Table 2.

However, there were no statistically significant differences in the distribution of genotypes and alleles between patients with PA and the reference group for the following SNPs: *TNKS2* rs10509639, *TERF2* rs251796, *ZNF676* rs412658, and *CTC1* rs3027234 (Table 2).

The Hardy–Weinberg equilibrium (HWE) test results demonstrated that genotypes of *TERF1* rs1545827, *TNKS2* rs10509637 and rs10509639, *TERF2* rs251796, *ZNF676* rs412658, and *CTC1* rs3027234 in the reference group did not deviate from HWE (*p* > 0.05). However, we identified that *TERF1* rs10107605 is not in HWE (Table 3). Regarding these findings, we excluded this SNP from the following analysis.

Binary logistic regression analysis was conducted in patients with PA and the reference group to investigate the associations of selected SNPs with PA occurrence. The results revealed the following associations. The *TERF1* rs1545827 CT + TT genotype, compared with CC, under the most robust genetic model (selected based on the lowest AIC value), is associated with 2.9-fold decreased odds of PA occurrence (OR 0.358; 95% CI: 0.235–0.546; *p* < 0.001). The *TNKS2* rs10509637 GG genotype, compared to AA, under the most robust recessive genetic model, is associated with 6.5-fold increased odds of PA occurrence (OR: 6.537; 95% CI: 3.015–14.172; *p* < 0.001) (Table 4).

However, no statistically significant results were found when analyzing associations between PA occurrence and *TNKS2* rs10509639, *TERF2* rs251796, *ZNF676* rs412658, and *CTC1* rs3027234.

The frequencies of genotypes and alleles for the selected SNPs were analyzed within the study groups, stratified by gender.

For *TERF1* rs1545827 (CC, CT, and TT), a statistically significant difference was observed between the PA and reference group females, with frequencies of 65.0%, 26.3%, and 8.7% in PA, respectively, compared to 37.1%, 48.9%, and 14.0% in the reference group (*p* < 0.001). Furthermore, the T allele was less frequent in the PA group, accounting for 21.9% compared to 38.5% in the reference group (*p* < 0.001). Similarly, for *TNKS2* rs10509637 (AA, AG, and GG), a significant difference was observed between the PA and reference groups, with frequencies of 52.5%, 31.3%, and 16.2% in PA, respectively, compared to 68.8%, 27.1%, and 4.1% in the reference group (*p* < 0.001). The G allele was more frequent in the PA group, accounting for 31.9% compared to 17.6% in the reference group (*p* < 0.001) (Table 5).

However, there were no statistically significant differences in the distribution of genotypes and alleles between females with PA and the reference group females for the following SNPs: *TNKS2* rs10509639, *TERF2* rs251796, *ZNF676* rs412658, and *CTC1* rs3027234.

Binary logistic regression analysis was conducted in patients with PA and the reference group to investigate the associations of selected SNPs with PA occurrence in females. The results revealed the following associations, The *TERF1* rs1545827 CC + TT genotype, compared with CC, under the most robust genetic model (selected based on the lowest AIC value), is associated with 3.1-fold decreased odds of PA occurrence in females (OR 0.318; 95% CI: 0.186–0.542; *p* < 0.001). The *TNKS2* rs10509637 AA genotype, compared to GG + AG, under the most robust recessive genetic model, is associated with 4.6-fold increased odds of PA occurrence in females (OR: 4.579; 95% CI: 1.871–11.165; *p* < 0.001). Additionally, the *TERF2* rs251796 AA genotype, compared to GG + AG, under the most robust recessive genetic model, is associated with 3-fold decreased odds of PA occurrence in females (OR: 0.335; 95% CI: 0.114–0.983; *p* = 0.047) (Table 6).

However, no statistically significant results were found when analyzing associations between PA occurrence in females and *TNKS2* rs10509639, *TERF2* rs251796, *ZNF676* rs412658, and *CTC1* rs3027234.

When analyzing males, we observed the following results. *TERF1* rs1545827 (CC, CT, and TT) statistically significantly differed between the PA and reference group males, with frequencies of 58.0%, 40.0%, and 2.0% in PA males, respectively, compared to 37.4%, 54.5%, and 8.1% in the reference group (*p* = 0.036). Furthermore, the T allele was less frequent in the PA group, accounting for 22.0% compared to 35.4% in the reference group males (*p* = 0.018). Additionally, for *TNKS2* rs10509637 (AA, AG, and GG), a significant difference was observed between the PA and reference group males, with frequencies of 50.0%, 32.0%, and 18.0% in males with PA, respectively, compared to 67.7%, 30.3%, and 2.0% in the reference group males (*p* = 0.001). The G allele was more frequent in the PA group males compared to the reference group males (34.0% vs. 7.2%, *p* = 0.001) (Table 7).

However, there were no statistically significant differences in the distribution of genotypes and alleles between males with PA and the reference group males for the following SNPs: *TNKS2* rs10509639, *TERF2* rs251796, *ZNF676* rs412658, and *CTC1* rs3027234.

Binary logistic regression analysis was conducted in patients with PA and the reference group to investigate the associations of selected SNPs with PA occurrence in males. The results revealed the following associations. The *TERF1* rs1545827 T allele, under the most robust genetic model (selected based on the lowest AIC value), is associated with 2.2-fold decreased odds of PA occurrence in males (OR 0.450; 95% CI: 0.242–0.834; *p* = 0.011). The *TNKS2* rs10509637 AA genotype, compared to GG + AG, under the most robust recessive genetic model, is associated with 10.6-fold increased odds of PA occurrence in males (OR: 10.646; 95% CI: 2.204–51.433; *p* = 0.003) (Table 8).

However, no statistically significant results were found when analyzing associations between PA occurrence in males and *TNKS2* rs10509639, *TERF2* rs251796, *ZNF676* rs412658, and *CTC1* rs3027234.

*TERF1* rs1545827, *TNKS2* rs10509637 and rs10509639, *TERF2* rs251796, *ZNF676* rs412658, and *CTC1* rs3027234 genes’ single nucleotide polymorphisms were analyzed to evaluate the associations with pituitary adenoma relapse. Only two SNPs, *TERF1* rs1545827 and *TNKS2* rs10509637, showed statistically significant results between the groups. Analyzing *TERF1* rs1545827, we found statistically significant results between PA without relapse compared with the reference group (CC, CT, and TT: 67.4%, 27.2%, and 5.4% vs. 37.2%, 50.6%, and 12.2%, respectively, *p* < 0.001); also, the T allele is statistically significantly less frequent in PA without relapse compared to the reference group (19.0% vs. 37.5%, *p* < 0.001). *TNKS2* rs10509637 showed statistically significant results between PA with relapse compared with the reference group (AA, AG, and GG: 52.6%, 34.2%, and 13.2% vs. 68.5%, 28.1%, and 3.4%, respectively, *p* = 0.012), and the G allele is statistically significantly more frequent in PA with relapse compared to the reference group (30.3% vs. 17.5%, *p* = 0.007). *TNKS2* rs10509637 showed statistically significant results between PA without relapse compared with the reference group (AA, AG, and GG: 51.1%, 30.4%, and 18.5% vs. 68.5%, 28.1%, and 3.4%, respectively, *p* < 0.001), and the G allele is statistically significantly more frequent in PA with relapse compared to the reference group (33.7% vs. 17.5%, *p* < 0.001) (Table 9).

Binary logistic regression analysis was conducted in patients with pituitary adenoma (PA) with or without relapse, as well as in the reference group. The results revealed the following associations. The *TNKS2* rs10509637 AA genotype, compared with GG + AG, under the most robust genetic model (selected based on the lowest AIC value), is associated with 4.2-fold increased odds of PA with relapse occurrence (OR 4.256; 95% CI: 1.394–12.998; *p* = 0.011). The *TERF1* rs1545827 CT + TT genotype, compared with CC, under the most robust genetic model, is associated with 3.5-fold decreased odds of PA without relapse occurrence (OR 0.286; 95% CI: 0.175–0.468; *p* < 0.001). The *TNKS2* rs10509637 AA genotype, compared to GG + AG, under the most robust recessive genetic model, is associated with 6.4-fold increased odds of PA without relapse occurrence (OR: 6.367; 95% CI: 2.863–14.160; *p* < 0.001) (Table 10).

However, no statistically significant results were found when analyzing associations between PA with relapse and *TERF1* rs1545827, *TNKS2* rs10509639, *TERF2* rs251796, *ZNF676* rs412658, and *CTC1* rs3027234. Additionally, no significant associations were observed between PA without relapse and *TNKS2* rs10509639, *TERF2* rs251796, *ZNF676* rs412658, and *CTC1* rs3027234.

**Figure 2:**
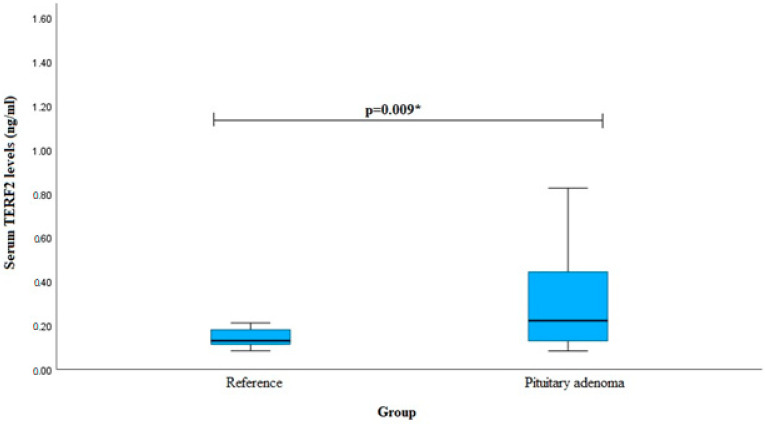
Serum TERF2 levels in PA and reference groups. * Mann–Whitney U-test was used.

Serum TERF2 levels were measured in patients with pituitary adenoma (*n* = 40) and the reference group (*n* = 40). We found that PA patients had elevated TERF2 serum levels when compared to the reference group (median (IQR): 0.222 (0.326) vs. 0.131 (0.072), *p* = 0.009). The results are shown in Figure 2.

**Figure 3:**
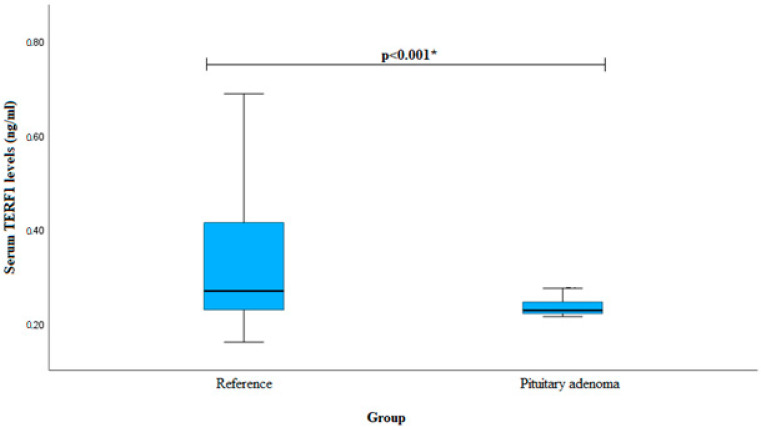
Serum TERF1 levels in PA and reference groups. * Mann–Whitney U-test was used.

Serum TERF1 levels were measured in patients with pituitary adenoma (*n* = 40) and the reference group (*n* = 60). We found that PA patients had decreased TERF1 serum levels when compared to the reference group (median (IQR): 0.227 (0.027) vs. 0.269 (0.195), *p* < 0.001). The results are shown in Figure 3.

**Figure 4:**
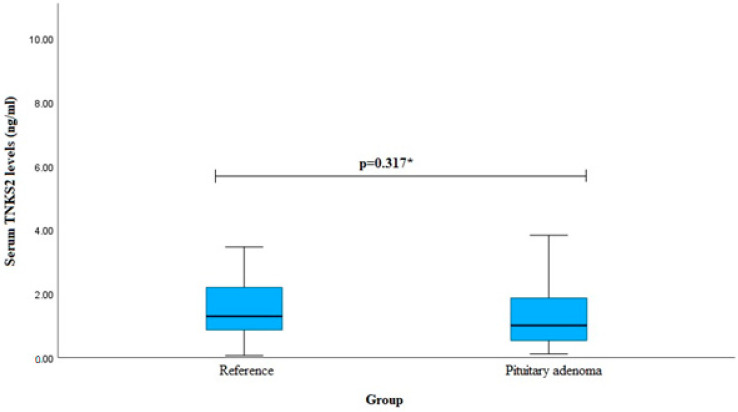
Serum TNKS2 levels in PA and reference groups. * Mann–Whitney U-test was used.

Serum TNKS2 levels were measured in patients with pituitary adenoma (*n* = 40) and reference (*n* = 40) groups, but no statistically significant difference was found (median (IQR): 1.001 (1.359) vs. 1.293 (1.382), *p* = 0.317). The results are shown in Figure 4.

**Figure 5:**
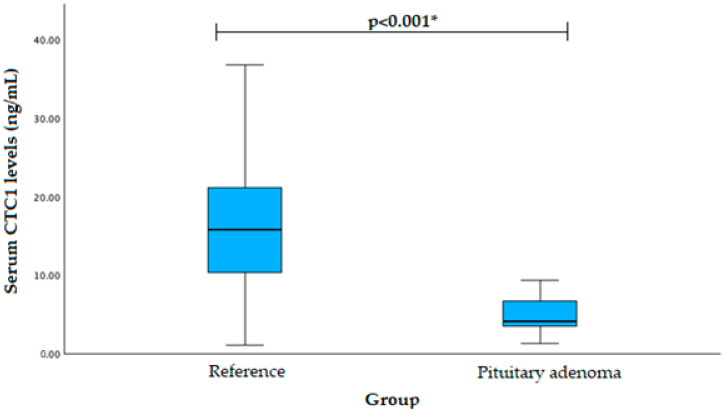
Serum CTC1 levels in PA and reference groups. * Student’s *t*-test was used.

Serum CTC1 levels were measured in patients with pituitary adenoma (*n* = 40) and the reference group (*n* = 40). We found that PA patients had decreased CTC1 serum levels when compared to the reference group (mean (SD): 6.155 (6.876) vs. 16.356 (8.409), *p* < 0.001). The results are shown in Figure 5.

**Figure 6:**
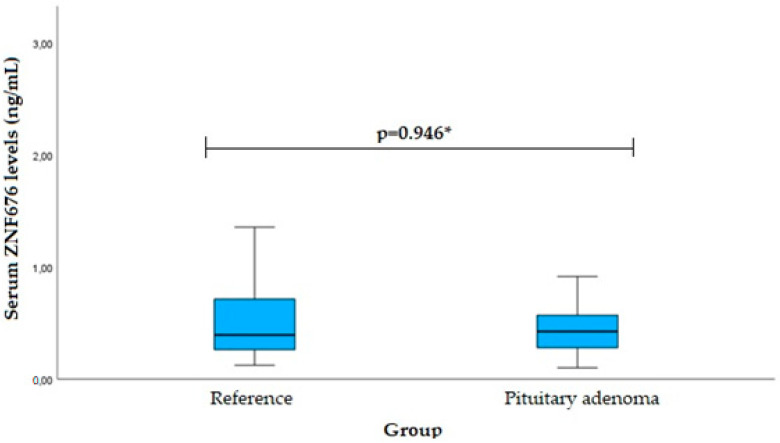
Serum ZNF676 levels in PA and reference groups. * Mann–Whitney U-test was used.

Serum TNKS2 levels were measured in patients with pituitary adenoma (*n* = 40) and reference (*n* = 40) groups, but no statistically significant difference was found (median (IQR): 0.426 (0.327) vs. 0.394 (0.455), *p* = 0.946). The results are shown in Figure 6.

**Figure 7:**
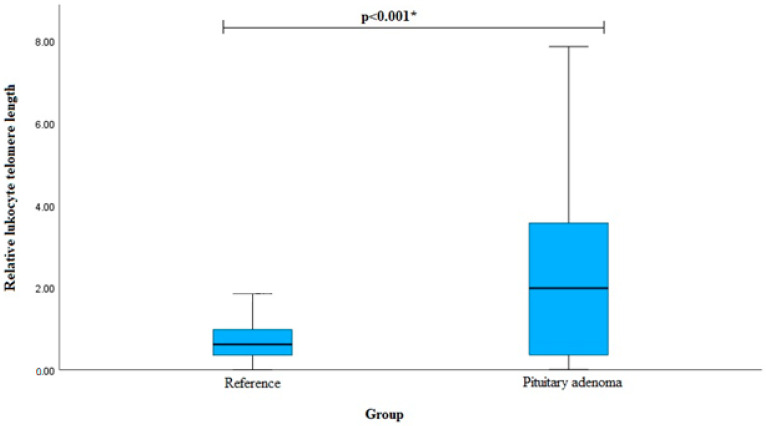
Relative leukocyte telomere length between PA and reference groups. * Mann–Whitney U-test was used.

Relative leukocyte telomere length (RLTL) was measured for 100 PA patients and 320 reference group subjects. We found a statistically significant difference in RLTL between the PA group and the reference group (median (IQR): 1.987 (3.225) vs. 0.619 (0.632), *p* < 0.001). The results are shown in Figure 7.

Regarding the median length of the reference groups’ RLTL, we performed an analysis of subjects with long telomeres (when RLTL ≥ 0.619) and those with short telomeres (when RLTL < 0.619). For *TERF1* rs1545827 (CC, CT, and TT), a statistically significant difference was observed between the long and short telomeres, with frequencies of 46.8%, 45.0%, and 8.2% in long telomeres, respectively, compared to 36.9%, 48.7%, and 14.4% in the short telomere group (*p* = 0.043). Furthermore, the T allele was less frequent in the long telomere group compared to the short telomere group (30.1% vs. 38.8%, *p* = 0.015). For *CTC1* rs3027234 (CC, CT, and TT), a statistically significant difference was observed between the long and short telomeres, with frequencies of 57.1%, 34.2%, and 8.7% in long telomeres, respectively, compared to 67.9%, 29.9%, and 2.1% in the short telomere group (*p* = 0.006). Furthermore, the T allele was more frequent in the long telomere group compared to the short telomere group (25.8% vs. 17.1%, *p* = 0.003) (Table 11).

Binary logistic regression analysis of telomere shortening was conducted. The results revealed the following associations. The *TERF1* rs1545827 T allele, under the most robust genetic model, is associated with 1.4-fold decreased odds of telomere shortening (OR 0.690; 95% CI: 0.513–0.927; *p* = 0.014). The *CTC1* rs3027234 TT genotype, compared to CC, under the most robust recessive genetic model, is associated with 4.8-fold increased odds of telomere shortening (OR: 4.336; 95% CI: 1.456–12.919; *p* = 0.008) (Table 12).

However, no statistically significant results were found when analyzing associations between telomere length and *TNKS2* rs10509639, rs10509637, *TERF2* rs251796, and *ZNF676* rs412658.

## 4. Discussion

The maintenance of chromosome integrity heavily relies on telomere dynamics. Alterations in telomere length can potentially contribute to the development of cancer [20]. Leukocyte telomere length is known to be heritable and has been associated with longevity. However, diverse findings regarding the heritability of telomere length and the impact of telomere biology on longevity have emerged in various populations [21].

In our study, we aimed to investigate whether genetic variations in genes related to telomere maintenance are linked to telomere length and influence the occurrence of PA. To address this, we analyzed seven polymorphisms within five telomerase-associated genes: *TNKS2* rs10509639 ir rs10509637, *CTC1* rs3027234, *ZNF676* rs412658, *TERF1* rs10107605 and rs1545827, *TERF2* rs251796. As a result, we have unveiled genetic correlations and molecular markers associated with PA incidence, gender-specific effects, and PA recurrence. Notably, the *TNKS2* rs10509637 GG genotype showed 6.5-fold increased odds of PA occurrence, suggesting a potential susceptibility factor. Conversely, the *TERF1* rs1545827 CT + TT genotypes were associated with 2.9-fold reduced odds of PA occurrence, indicating a protective genetic factor. We observed parallels with the findings of Varadi et al., who investigated similar genetic associations. Their research demonstrated that G allele carriers of rs10509637 were associated with an increased risk for breast cancer (OR 1.33, 95% CI 1.08–1.62) [22]. These findings emphasize the interplay of genetics in disease susceptibility and may provide valuable insights into the pathogenesis of PA. However, it is essential to emphasize that, while we observed statistically significant associations between the *TERF1* rs1545827 and decreased odds of PA occurrence, there is a lack of studies reporting the same results in relation to oncogenesis.

The decision to conduct separate analyses for females and males aimed to investigate potential gender-specific effects on genetic associations with PA occurrence. Our analysis showed that the *TERF1* rs1545827 CC + TT genotype was associated with 3.1-fold lower odds of PA occurrence in females and 2.2-fold lower odds in males, whereas the *TNKS2* rs10509637 AA genotype was linked to 4.6-fold higher odds in females and, astonishingly, 10.6-fold higher odds in males. These findings underscore the presence of gender-specific genetic influences, particularly in the case of *TERF1* rs1545827, while *TNKS2* displayed increased odds in both females and males.

Exploring the recurrence patterns of PA and the genetic factors associated with them holds the potential to improve remission rates [23]. This knowledge enables a more targeted approach to early diagnosis and the development of enhanced treatment strategies. Consequently, we conducted an analysis focused on PA recurrence, revealing that the *TNKS2* rs10509637 AA genotype was associated with 4.2-fold increased odds of PA recurrence. Notably, Salhab and colleagues, in their study, demonstrated a decreased expression of the *TNKS2* gene as breast cancer progression advanced [24]. This highlights the complex role of TNKS2 in different diseases and underscores the significance of further research to unravel its multifaceted functions.

Additionally, our study suggests a potential role for TERF1, TERF2, and CTC1 as biomarkers and indicators in the context of PA. While elevated TERF2 levels in serum may be indicative of PA, the lower levels of serum TERF1 and CTC1 observed in PA patients provide valuable insights into the underlying pathophysiology of this condition. However, previous research, as highlighted by Bhari et al., has linked high expression levels of both TERF1 and TERF2 to a poor prognosis in breast cancer, suggesting their potential as prognostic markers in cancer [25]. Additionally, the work of Marcos and colleagues has indicated that TERF1 is deregulated in the context of cancer development, underscoring its relevance in the broader field of oncology [26].

Our study did not reveal any statistically significant difference between *ZNF676* gene polymorphism and ZNF676 serum levels and patients with PA. de Araújo at al. confirmed the overexpression of ZNF676 (mean increase of 5.13-fold in invasive and 2.04-fold in non-invasive corticotrophinomas compared to the calibrator), and overexpression of ZNF676 in patients in the invasive group had a higher mean preoperative ACTH level (102.3 ± 52.2 pg/mL, normal range 50–310 μg/24 h) compared to patients in the non-invasive group (51.7 ± 15.9 pg/mL, normal range < 46 pg/mL). In contrast, patients with non-invasive corticotrophinomas had higher urinary cortisol concentrations (639.6 ± 358.0 μg/24 h) than patients with invasive corticotrophinomas (406.0 ± 34.8 μg/24 h) [16].

The observed variations in the levels of TERF1, TERF2, and CTC1 in different medical contexts underscore the complex roles these telomere-related proteins play in various diseases. Future studies should further investigate the mechanistic connections between these proteins and the pathogenesis of PA, as well as their potential applications in cancer diagnosis and prognosis.

Furthermore, our study revealed a statistically significant difference in relative leukocyte telomere length between PA patients and the reference group (*p* < 0.001). This finding aligns with the results of a study conducted by Heaphy et al., which also reported the prevalence of short telomeres in PAs [27].

Exploring genetic correlations with telomere shortening deepens our comprehension of telomere maintenance. For example, in our study, the *TERF1* rs1545827 T allele displays a protective influence on telomere shortening (*p* = 0.014). This observation aligns with the role of *TERF1* as a suppressor of telomere elongation, suggesting its involvement in a negative feedback mechanism that stabilizes telomere length [28]. Our study also indicates a significant association between the *CTC1* rs3027234 TT genotype and accelerated telomere shortening (*p* = 0.008). These findings align with the work of Mangino et al., who also observed that the rs3027234 T allele was linked to shorter telomere length [13]. The consistent evidence of this genetic variant’s impact on telomere dynamics strengthens the understanding of telomere maintenance and suggests that the *CTC1* gene may play a role in regulating telomere length. This insight underscores the importance of genetic factors in influencing telomere homeostasis, potentially opening new avenues for targeted interventions in diseases related to telomere dysfunction.

The complex of genetics, gender-specific influences, telomere dynamics, and serum indicators in PA pathogenesis is clarified by these findings. To understand the underlying mechanisms and their therapeutic implications, more research is necessary.

## 5. Conclusions

In conclusion, our study underscores the intricate interplay between genetic factors, telomere dynamics, and pituitary adenoma susceptibility. The associations observed with *TERF1* and *TNKS2* genes suggest their potential roles in telomere regulation and disease predisposition.

## Figures and Tables

**Table 1:** Demographic characteristics of the study.

Characteristics		Group		*p*-Value
		PA Group	Reference Group	
Gender	Males, *n* (%)	50 (38.5)	99 (30.9)	0.124
	Females, *n* (%)	80 (61.5)	221 (69.1)	
Age Mean (SD)		52.73 (14.118)	51.88 (21.325)	0.620 *
Relative leukocyte telomere length Median (IQR)		1.987 (3.225)	0.619 (0.632)	<0.001 **

* Student’s *t*-test was used; ** Mann–Whitney U-test was used; PA—pituitary adenoma; SD—Std. Deviation; IQR—Interquartile Range; *p*-value: significance level (alpha = 0.05).

**Table 2:** Genotype and allele frequencies of single nucleotide polymorphisms (*TERF1* rs1545827 and rs10107605, *TNKS2* rs10509637 and rs10509639, *TERF2* rs251796, *ZNF676* rs412658, *CTC1* rs3027234) within PA and reference groups.

Gene, SNP	Genotype, Allele	PA Group, *n* (%)	Reference Group, *n* (%)	*p*-Value
*TERF1* rs1545827	CC	81 (62.3)	119 (37.2)	<0.001
	CT	41 (31.5)	162 (50.6)	
	TT	8 (6.2)	39 (12.2)	
	Total	130 (100)	320 (100)	
	Allele			
	C	203 (78.1)	400 (62.5)	<0.001
	T	57 (21.9)	240 (37.5)	
*TERF1* rs10107605	AA	118 (90.8)	259 (80.9)	0.007
	AC	12 (9.2)	42 (13.1)	
	CC	0 (0.0)	19 (5.9)	
	Total	130 (100)	320 (100)	
	Allele			
	A	248 (95.4)	560 (87.5)	<0.001
	C	12 (4.6)	80 (12.5)	
*TNKS2* rs10509637	AA	67 (51.6)	219 (68.5)	<0.001
	AG	41 (31.5)	90 (28.1)	
	GG	22 (16.8)	11 (3.4)	
	Total	130 (100)	320 (100)	
	Allele			
	A	175 (67.3)	528 (82.5)	<0.001
	G	85 (32.7)	112 (17.5)	
*TNKS2* rs10509639	AA	107 (82.3)	252 (78.8)	0.513
	AG	22 (16.9)	67 (20.9)	
	GG	1 (0.8)	1 (0.3)	
	Total	130 (100)	320 (100)	
	Allele			
	A	236 (90.8)	571 (89.2)	0.489
	G	24 (9.2)	69 (10.8)	
*TERF2* rs251796	AA	61 (46.9)	154 (48.1)	0.215
	AG	59 (45.4)	125 (39.1)	
	GG	10 (7.7)	41 (12.8)	
	Total	130 (100)	320 (100)	
	Allele			
	A	181 (69.6)	433 (67.7)	0.567
	G	79 (30.4)	207 (32.3)	
*ZNF676* rs412658	CC	64 (49.2)	135 (42.2)	0.287
	CT	50 (38.5)	149 (46.6)	
	TT	16 (12.3)	36 (11.2)	
	Total	130 (100)	320 (100)	
	Allele			
	C	178 (68.5)	419 (65.5)	0.389
	T	82 (31.5)	221 (34.5)	
*CTC1* rs3027234	CC	78 (60.0)	199 (62.2)	0.702
	CT	46 (35.4)	102 (31.9)	
	TT	6 (4.6)	19 (5.9)	
	Total	130 (100)	320 (100)	
	Allele			
	C	202 (76.9)	500 (78.1)	0.887
	T	58 (23.1)	140 (21.9)	

**Table 3:** Analysis of Hardy–Weinberg equilibrium in the reference group.

Gene and SNP	Allele Frequencies		Genotype Distribution	*p*-Value
*TERF1* rs1545827	0.62 C	0.38 T	39/162/119	0.152
*TERF1* rs10107605	0.87 A	0.13 C	19/42/259	<0.0001
*TNKS2* rs10509637	0.82 A	0.18 G	11/90/219	0.642
*TNKS2* rs10509639	0.89 A	0.11 G	1/67/252	0.114
*TERF2* rs251796	0.68 A	0.32 G	41/125/154	0.055
*ZNF676* rs412658	0.65 C	0.35 T	36/149/135	0.594
*CTC1* rs3027234	0.78 C	0.22 T	19/102/199	0.228

SNP—Single nucleotide polymorphism; *p*-value—significance level (alpha = 0.05).

**Table 4:** Binary logistic regression analysis within patients with pituitary adenoma and reference group subjects.

Model	Genotype/Allele	OR (95% CI)	*p*-Value	AIC
*TERF1 rs1545827*				
Codominant	CT vs. CC TT vs. CC	0.372 (0.239–0.580) 0.301 (0.134–0.678)	**<0.001** **0.004**	521.1
Dominant	CT + TT vs. CC	0.358 (0.235–0.546)	**<0.001**	519.4
Recessive	TT vs. CC + CT	0.472 (0.214–1.041)	0.063	519.1
Overdominant	CT vs. CC + TT	0.449 (0.292–0.691)	**<0.001**	529.2
Additive	T	0.453 (0.320–0.642)	**<0.001**	521.1
*TNKS2 rs10509637*				
Codominant	AG vs. AA GG vs. AA	1.489 (0.940–2.358) 6.537 (3.015–14.172)	0.090 **<0.001**	520.2
Dominant	AG + GG vs. AA	2.039 (1.344–3.094)	**<0.001**	531.9
Recessive	AA vs. GG + AG	5.722 (2.686–12.189)	**<0.001**	521.1
Overdominant	AG vs. AA + GG	1.177 (0.756–1.833)	0.470	542.5
Additive	G	2.081 (1.516–2.857)	**<0.001**	522.3
*TNKS2 rs10509639*				
Codominant	AG vs. AA GG vs. AA	0.773 (0.454–1.317) 2.355 (0.146–38.001)	0.344 0.546	541.7
Dominant	AG + GG vs. AA	0.797 (0.472–1.345)	0.395	542.3
Recessive	AA vs. GG + AG	2.473 (0.154–39.834)	0.523	542.6
Overdominant	AG vs. AA + GG	0.769 (0.452–1.309)	0.333	540.1
Additive	G	0.831 (0.502–1.378)	0.474	542.5
*TERF2 rs251796*				
Codominant	AG vs. AA GG vs. AA	1.192 (0.776–1.829) 0.616 (0.290–1.306)	0.423 0.206	541.8
Dominant	AG + GG vs. AA	1.049 (0.698–1.579)	0.817	543.0
Recessive	AA vs. GG + AG	0.567 (0.275–1.169)	0.124	540.5
Overdominant	AG vs. AA + GG	1.296 (0.859–1.957)	0.217	541.5
Additive	G	0.917 (0.677–1.243)	0.578	542.7
*ZNF676 rs412658*				
Codominant	CT vs. CC TT vs. CC	0.708 (0.457–1.096) 0.938 (0.485–1.813)	0.121 0.848	542.5
Dominant	CT + TT vs. CC	0.753 (0.500–1.133)	0.173	541.2
Recessive	TT vs. CC + CT	1.107 (0.591–2.074)	0.750	542.9
Overdominant	CT vs. CC + TT	0.717 (0.473–1.087)	0.117	540.6
Additive	T	0.874 (0.643–1.189)	0.392	542.3
*CTC1 rs3027234*				
Codominant	CT vs. CC TT vs. CC	1.151 (0.744–1.779) 0.806 (0.310–2.093)	0.528 0.657	544.3
Dominant	CT + TT vs. CC	1.096 (0.722–1.664)	0.666	542.9
Recessive	TT vs. CC + CT	0.767 (0.299–1.965)	0.580	542.7
Overdominant	CT vs. CC + TT	1.170 (0.762–1.798)	0.473	542.5
Additive	T	1.024 (0.729–1.439)	0.889	543.0

PA—pituitary adenoma; OR: odds ratio; CI: confidence interval; *p* value: significance level (alpha = 0.05); AIC: Akaike information criterion. Statistically significant results marked in bold. The most robust genetic model underlined (selected based on the lowest AIC value).

**Table 5:** Genotype and allele frequencies of *TERF1* rs1545827, *TNKS2* rs10509637 within PA and reference group females.

Gene, SNP	Genotype, Allele	PA Group Females, *n* (%)	Reference Group Females, *n* (%)	*p*-Value
*TERF1* rs1545827	CC	52 (65.0)	82 (37.1)	<0.001
	CT	21 (26.3)	108 (48.9)	
	TT	7 (8.7)	31 (14.0)	
	Total	80 (100)	221 (100)	
	Allele			
	C	125 (78.1)	272 (61.5)	<0.001
	T	35 (21.9)	170 (38.5)	
*TNKS2* rs10509637	AA	42 (52.5)	152 (68.8)	<0.001
	AG	25 (31.3)	60 (27.1)	
	GG	13 (16.2)	9 (4.1)	
	Total	80 (100)	221 (100)	
	Allele			
	A	109 (68.1)	364 (82.4)	<0.001
	G	51 (31.9)	78 (17.6)	

**Table 6:** Binary logistic regression analysis within females with pituitary adenoma and reference group females.

Model	Genotype/Allele	OR (95% CI)	p-Value	AIC
*TERF1 rs1545827*				
Codominant	CT vs. CC TT vs. CC	0.307 (0.171–0.549) 0.356 (0.146–0.868)	**<0.001** **0.023**	333.9
Dominant	CT + TT vs. CC	0.318 (0.186–0.542)	**<0.001**	332.0
Recessive	TT vs. CC + CT	0.588 (0.248–1.394)	0.228	349.0
Overdominant	CT vs. CC + TT	0.372 (0.212–0.654)	**<0.001**	337.8
Additive	T	0.455 (0.298–0.697)	**<0.001**	335.9
*TNKS2 rs10509637*				
Codominant	AG vs. AA GG vs. AA	1.508 (0.846–2.689) 5.228 (2.092–3.065)	0.164 **<0.001**	359.5
Dominant	AG + GG vs. AA	1.993 (1.181–3.362)	**0.010**	343.9
Recessive	AA vs. GG + AG	4.579 (1.871–11.165)	**<0.001**	339.4
Overdominant	AG vs. AA + GG	1.220 (0.698–2.131)	0.485	350.1
Additive	G	1.973 (1.334–2.920)	**<0.001**	339.0
*TERF2 rs251796*				
Codominant	AG vs. AA GG vs. AA	0.917 (0.537–1.567) 0.322 (0.107–0.971)	0.752 **0.044**	347.5
Dominant	AG + GG vs. AA	0.765 (0.458–1.277)	**0.306**	349.5
Recessive	AA vs. GG + AG	0.335 (0.114–0.983)	**0.047**	345.6
Overdominant	AG vs. AA + GG	1.081 (0.643–1.820)	0.768	350.5
Additive	G	0.707 (0.476–1.051)	0.086	347.5

PA—pituitary adenoma; OR: odds ratio; CI: confidence interval; *p* value: significance level (alpha = 0.05); AIC: Akaike information criterion. Statistically significant results marked in bold. The most robust genetic model underlined (selected based on the lowest AIC value).

**Table 7:** Genotype and allele frequencies of single nucleotide polymorphisms *TERF1* rs1545827 and *TNKS2* rs10509637 within PA and reference group males.

Gene, SNP	Genotype, Allele	PA Group Males, *n* (%)	Reference Group Males, *n* (%)	*p*-Value
*TERF1* rs1545827	CC	29 (58.0)	37 (37.4)	0.036
	CT	20 (40.0)	54 (54.5)	
	TT	1 (2.0)	8 (8.1)	
	Total	50 (100)	99 (100)	
	Allele			
	C	78 (78.0)	128 (64.6)	0.018
	T	22 (22.0)	70 (35.4)	
*TNKS2* rs10509637	AA	25 (50.0)	67 (67.7)	0.001
	AG	16 (32.0)	30 (30.3)	
	GG	9 (18.0)	2 (2.0)	
	Total	50 (100)	99 (100)	
	Allele			
	A	66 (66.0)	164 (82.8)	0.001
	G	34 (34.0)	34 (17.2)	

**Table 8:** Binary logistic regression analysis within males with pituitary adenoma and reference group males.

Model	Genotype/Allele	OR (95% CI)	*p*-Value	AIC
*TERF1 rs1545827*				
Codominant	CT vs. CC TT vs. CC	0.473 (0.233–0.958) 0.159 (0.019–1.349)	**0.038** 0.092	187.2
Dominant	CT + TT vs. CC	0.432 (0.216–0.865)	**0.018**	186.4
Recessive	TT vs. CC + CT	0.232 (0.028–1.910)	0.174	189.6
Overdominant	CT vs. CC + TT	0.556 (0.279–1.108)	0.095	189.3
Additive	T	0.450 (0.242–0.834)	**0.011**	185.2
*TNKS2 rs10509637*				
Codominant	AG vs. AA GG vs. AA	1.489 (0.668–3.059) 2.060 (2.436–59.707)	0.358 **0.002**	181.5
Dominant	AG + GG vs. AA	2.094 (1.044–4.200)	**0.037**	187.8
Recessive	AA vs. GG + AG	10.646 (2.204–51.433)	**0.003**	180.3
Overdominant	AG vs. AA + GG	1.082 (0.520–2.252)	0.832	192.1
Additive	G	2.298 (1.330–3.972)	**0.003**	182.9

PA—pituitary adenoma; OR: odds ratio; CI: confidence interval; *p* value: significance level (alpha = 0.05); AIC: Akaike information criterion. Statistically significant results marked in bold. The most robust genetic model underlined (selected based on the lowest AIC value).

**Table 9:** *TERF1* rs1545827 and *TNKS2* rs10509637 genotype and allele frequencies within pituitary adenoma with relapse or without relapse and reference groups.

Gene, SNP	Genotype, Allele	Reference Group, *n* (%)	PA Group with Relapse, *n* (%)	*p*-Value	PA Group without Relapse, *n* (%)	*p*-Value
*TERF1* rs1545827	CC	119 (37.2)	19 (50.0)	0.290	62 (67.4)	<0.001
	CT	162 (50.6)	16 (42.1)		25 (27.2)	
	TT	39 (12.2)	3 (7.9)		5 (5.4)	
	Total	320 (100)	38 (100)		92 (100)	
	Allele					
	C	400 (62.5)	54 (71.1)	0.143	149 (81.0)	<0.001
	T	240 (37.5)	22 (28.9)		35 (19.0)	
*TNKS2* rs10509637	AA	219 (68.5)	20 (52.6)	0.012	47 (51.1)	<0.001
	AG	90 (28.1)	13 (34.2)		28 (30.4)	
	GG	11 (3.4)	5 (13.2)		17 (18.5)	
	Total	320 (100)	38 (100)		92 (100)	
	Allele					
	A	528 (82.5)	53 (69.7)	0.007	122 (66.3)	<0.001
	G	112 (17.5)	23 (30.3)		62 (33.7)	

**Table 10:** Binary logistic regression analysis within PA with or without relapse and reference group subjects.

Model	Genotype/Allele	OR (95% CI)	*p*-Value	AIC
**PA with Relapse**				
*TNKS2* rs10509637				
Codominant	AG vs. AA GG vs. AA	1.582 (0.755–3.316) 4.977 (1.573–15.751)	0.225 **0.006**	239.5
Dominant	AG + GG vs. AA	1.951 (0.990–3.848)	0.054	240.6
Recessive	AA vs. GG + AG	4.256 (1.394–12.998)	**0.011**	238.9
Overdominant	AG vs. AA + GG	1.329 (0.651–2.711)	0.435	243.7
Additive	G	1.968 (1.170–3.311)	**0.011**	238.2
**PA without relapse**				
*TERF1* rs1545827				
Codominant	CT vs. CC TT vs. CC	0.296 (0.176–0.499) 0.246 (0.092–0.656)	**<0.001** **0.005**	414.9
Dominant	CT + TT vs. CC	0.286 (0.175–0.468)	**<0.001**	413.1
Recessive	TT vs. CC + CT	0.414 (0.158–1.083)	0.072	436.7
Overdominant	CT vs. CC + TT	0.364 (0.219–0.605)	**<0.001**	423.1
Additive	T	0.375 (0.247–0.570)	**<0.001**	415.2
*TNKS2* rs10509637				
Codominant	AG vs. AA GG vs. AA	1.450 (0.855–2.459) 7.201 (3.168–16.371)	0.168 **<0.001**	418.9
Dominant	AG + GG vs. AA	2.076 (1.295–3.328)	**0.002**	430.5
Recessive	AA vs. GG + AG	6.367 (2.863–14.160)	**<0.001**	418.8
Overdominant	AG vs. AA + GG	1.118 (0.674–1.855)	0.666	439.4
Additive	G	2.173 (1.526–3.096)	**<0.001**	421.3

PA—pituitary adenoma; OR: odds ratio; CI: confidence interval; *p* value: significance level (alpha = 0.05); AIC: Akaike information criterion. Statistically significant results marked in bold. The most robust genetic model underlined (selected based on the lowest AIC value).

**Table 11:** Frequencies of genotypes and alleles of *TERF1* rs1545827 and *CTC1* rs3027234 in the long and short telomere groups (T/S median = 0.619).

Gene, SNP	Genotype, Allele	Long Telomeres	Short Telomeres	*p*-Value
*TERF1* rs1545827	CC	108 (46.8)	69 (36.9)	0.043
	CT	104 (45.0)	91 (48.7)	
	TT	19 (8.2)	27 (14.4)	
	Total	231 (100)	187 (100)	
	Allele			
	C	320 (69.3)	229 (61.2)	0.015
	T	142 (30.1)	145 (38.8)	
*CTC1* rs3027234	CC	132 (57.1)	127 (67.9)	0.006
	CT	79 (34.2)	56 (29.9)	
	TT	20 (8.7)	4 (2.1)	
	Total	231 (100)	187 (100)	
	Allele			
	C	343 (74.2)	310 (82.9)	0.003
	T	119 (25.8)	64 (17.1)	

**Table 12:** Binary logistic regression analysis of *TERF1* rs1545827 and *CTC1* rs3027234 in telomere shortening.

Model	Genotype/Allele	OR (95% CI)	*p*-Value	AIC
*TERF1* rs1545827				
Codominant	CT vs. CC TT vs. CC	0.730 (0.483–1.103) 0.450 (0.232–0.870)	0.135 **0.018**	572.5
Dominant	CT + TT vs. CC	0.666 (0.449–0.987)	**0.043**	572.7
Recessive	TT vs. CC + CT	0.531(0.285–0.989)	**0.046**	572.8
Overdominant	CT vs. CC + TT	0.864 (0.587–1.272)	0.458	576.3
Additive	T	0.690 (0.513–0.927)	**0.014**	570.7
*CTC1* rs3027234				
Codominant	CT vs. CC TT vs. CC	1.357 (0.892–2.066) 4.811 (1.600–14.464)	0.154 **0.005**	565.8
Dominant	CT + TT vs. CC	1.587 (1.061–2.375)	**0.024**	571.7
Recessive	TT vs. CC + CT	4.336 (1.456–12.919)	**0.008**	567.8
Overdominant	CT vs. CC + TT	1.216 (0.803–1.840)	0.355	576.0
Additive	T	1.644 (1.174–2.302)	**0.004**	568.1

PA—pituitary adenoma; OR: odds ratio; CI: confidence interval; *p* value: significance level (alpha = 0.05); AIC: Akaike information criterion. Statistically significant results marked in bold. The most robust genetic model underlined (selected based on the lowest AIC value).

## Data Availability

The data can be shared up on request.
